# Sodium Ascorbate as a Quorum-Sensing Inhibitor Leads to Decreased Virulence in *Vibrio campbellii*

**DOI:** 10.3389/fmicb.2020.01054

**Published:** 2020-06-05

**Authors:** Biao Han, Xiaoting Zheng, Kartik Baruah, Peter Bossier

**Affiliations:** ^1^Laboratory of Aquaculture & Artemia Reference Center, Department of Animal Sciences and Aquatic Ecology, Faculty of Bioscience Engineering, Ghent University, Ghent, Belgium; ^2^Department of Animal Nutrition and Management, Faculty of Veterinary Medicine and Animal Sciences, Swedish University of Agricultural Sciences, Uppsala, Sweden

**Keywords:** sodium ascorbate, quorum sensing inhibitor, virulence, *Vibrio campbellii*, *Artemia*

## Abstract

*Vibrio campbellii* is one of the major bacterial pathogens for animals reared in aquaculture, affecting both vertebrates and invertebrates, and causes significant economic losses. It is now evident that the expressions of virulence factors in this pathogen are regulated by the density of the bacterial population. This type of regulation, termed quorum sensing (QS), is mediated by extracellular signal molecules called autoinducers. In this study, the impact of sodium ascorbate (NaAs) on the virulence of *V. campbellii* was investigated under both *in vitro* and *in vivo* conditions, to develop a natural anti-infective strategy to contain *V. campbellii* infection in aquacultured animals. Results showed that NaAs significantly decreased swimming motility, biofilm production, and the production of virulence enzymes, such as lipase, caseinase, phospholipase, and hemolysin in *V. campbellii*. Consistent with this, pretreatment of *V. campbellii* with NaAs before inoculation into the rearing water resulted in significantly increased survival of gnotobiotic brine shrimp larvae, when compared to larvae challenged with untreated *V. campbellii*. Furthermore, NaAs could interfere with QS-regulated bioluminescence in *V. campbellii*, suggesting the QS-inhibitory activity largely determines the protective effect of NaAs toward the brine shrimp. In essence, due to the potent anti-virulence effects observed in *in vitro* studies and the clinical brine shrimp-*V. campbellii* infection model, NaAs constitute a promising novel strategy for the control of *V. campbellii* infections in aquaculture.

## Introduction

*Vibrio campbellii* is a Gram-negative luminous bacterium that lives in a broad range of aquatic environments. It has been regarded as a serious pathogen affecting numerous vertebrates and invertebrates, which leads to significant losses to the aquaculture industry (Defoirdt et al., 2008; Yang et al., 2015). Recent studies have shed some light on the pathogenicity mechanisms of this bacterial strain. In general, the infectious steps of bacterial pathogens include adhesion and incubation in the host, avoidance of host defenses, causing diseases and mortality, and then exit (Donnenberg, 2000). These steps involve the expression of virulence factors that allow the pathogens to infect and damage the host. For *Vibrio campbellii*, the ability to adhere to host surface and form biofilms, produce extracellular products (E) and lipopolysaccharide (LPS), and interact with bacteriophage and bacteriocin-like substance (BLIS) are its most crucial virulence determinants (Austin and Zhang, 2006; Darshanee Ruwandeepika et al., 2012).

**FIGURE 1:**
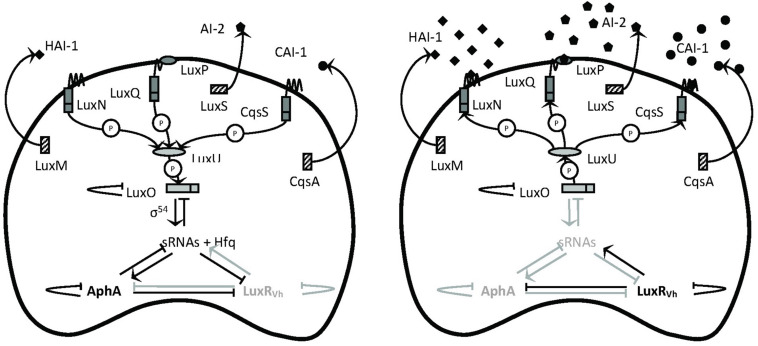
Schematic representation of the quorum-sensing circuit of *Vibrio campbellii*. The LuxM, LuxS, and CqsA enzymes synthesize the autoinducers HAI-1, AI-2, and CAI-1, respectively (Winzer et al., 2002). These autoinducers are detected at the cell surface by the LuxN, LuxPQ, and CqsS two-component hybrid sensor kinase/response regulator proteins, respectively. In complex of AI-2 by LuxQ requires the interacts with the periplasmic protein LuxP to send the AI-2 signal (Bassler et al., 1993). In the absence of autoinducers (left, at low cell density), the receptors autophosphorylate and transfer phosphate to LuxO via LuxU (Freeman and Bassler, 1999). Phosphorylation activates LuxO, which together with σ^54^ activates the production of five small regulatory RNAs (sRNAs). The sRNAs, together with the chaperone Hfq, promote translation of the master regulator AphA and inhibit translation of the master regulator LuxR_Vh_ (Tu and Bassler, 2007). In the presence of high concentrations of the autoinducers (right, at high cell density), the receptor proteins switch from kinases to phosphatases, which results in dephosphorylation of LuxO. Dephosphorylated LuxO is inactive and therefore, the sRNAs are not formed, AphA is not translated, and LuxR_Vh_ is translated. AphA and LuxR are transcriptional regulators that (either individually or together) affect the transcription of many target genes. P means phosphotransfer. Modified with permission from Defoirdt et al. (2008) and Yang et al. (2017).

Since virulence factors are often costly metabolic products, their expression usually is under tight control to avoid energy waste. One of the regulatory mechanisms controlling the production of virulence factors in *V. campbellii* is quorum-sensing (QS), cell-to-cell communication with secreted signaling molecules called autoinducers (Defoirdt et al., 2008). The external concentration of autoinducer varies according to the cell population density of the bacteria in the environment. When the autoinducer concentration reached the threshold, the bacteria could initiate a signal transduction cascade that culminates in a change in the behavior, such as controlling luminescence, biofilm formation, and toxin production, in response to environmental cues (Schauder and Bassler, 2001). The model describing the QS circuit employed in *V. campbellii* is presented in Figure 1. *V. campbellii* is a model bacterium in QS research and has been found to contain a three-channel QS system that uses three kinds of signaling molecules: campbellii autoinducer 1 (HAI-1) (Cao and Meighen, 1989), autoinducer 2 (AI-2) (Chen et al., 2002), and cholerae autoinducer 1 (CAI-1) (Higgins et al., 2007). Previous work in our lab had shown that the three-channel QS system is required for the full virulence of *V. campbellii* (Defoirdt and Sorgeloos, 2012; Pande et al., 2013).

Disrupting the QS signaling pathways has been proposed as a suitable strategy for the development of new antimicrobial and anti-virulence therapies. This strategy is highly attractive because it is unlikely to pose a selective pressure for the development of bacterial resistance (Rasko and Sperandio, 2010). Specifically, using the enzymes that degrade the autoinducers, a process called quorum quenching, and library screening for the analogs of autoinducers are the most used strategies to inhibit the organism-specific cell to cell signaling. To date, several QS inhibitors have been described. Amongst all, the halogenated furanones produced by the marine algae *Delisea pulchra* are the most studied QS inhibitors (Rasmussen et al., 2000; Manefield et al., 2001). These compounds, which are structurally similar to AHLs, were reported to disrupt QS in many Gram-negative bacteria by decreasing the DNA-binding activity of the QS master regulator *LuxR* (Manefield et al., 2002; Smith et al., 2003; Defoirdt et al., 2007). Unfortunately, these halogenated furanones are toxic *per se* to higher organisms (Wang et al., 2016), which limited their practical applications. However, their proven ability to control bacterial infections in animal models is of considerable importance, since it demonstrates that QS is a useful and promising target for antibacterial agents. Over the past years, several naturally occurring furanones have been explored as an alternative for the corresponding halogenated furanones. One of these compounds, the ascorbic acid [5-(1,2-dihydroxyethyl)-3,4-dihydroxy-2(5H)-furanone] or mineral ascorbates (sodium ascorbate), has been identified as interesting QS analogs because they tend to be relatively less invasive and highly effective in reducing the QS and pathogenic potential of many bacteria like *Pseudomonas aeruginosa* and *Clostridium perfringens* (Novak and Fratamico, 2004; El-Mowafy et al., 2014). Although these compounds show a high degree of QS interfering effects in the bacterial pathogens of humans, they were evaluated neither for their effect on QS-regulated virulence factors in aquaculture pathogens of commercial importance nor for their antibacterial activity *in vivo*. This study aimed at investigating the effect of NaAs on the virulence of *V. campbellii in vitro* and *in vivo* in a highly controlled model system with gnotobiotic brine shrimp (*Artemia franciscana*) larvae, and the results of this study could provide a novel strategy for the control of *V. campbellii* infections in aquaculture.

## Materials and Methods

### Bacterial Strains, Culture Media, and Reagents

*Vibrio campbellii* BAA-1116 wild type strain^1^, mutant strain JAF548 pAKlux1, and *Aeromonas* spp. strain LVS3 were used in this study. LVS3, after autoclaving, was used as feed for brine shrimp. The mutant strain contains a point mutation in *LuxO*, which renders the QS system inactive. And the plasmid pAKlux1 contains the *Photorhabdus luminescens* bioluminescence operon under the control of a constitutive promoter. Hence, the bioluminescence is independent of the three-channel QS system in this strain. All strains were preserved at −80°C in Marine Broth 2216 (Difco Laboratories, Detroit, MI, United States) with 25% sterile glycerol. The bacterial strains were initially grown at 28°C for 24 h on Marine Agar (Difco Laboratories, Detroit, MI, United States) and then to log phase in Marine Broth by incubation at 28°C with continuous shaking before use. Bacterial cell densities were determined spectrophotometrically (BioMérieux, Marcy L’Etoile, France) at 600 nm according to the McFarland standard, assuming that an optical density of 1 corresponding to 1.2 × 10^9^ cells ml^–1^. NaAs was purchased from VWR international (Leuven, Belgium).

### Bacterial Growth Assays

For the bacterial growth assays, *V. campbellii* was grown overnight in Marine Broth at 28°C. After that, the culture was diluted to an OD_600_ of 0.1 in fresh Marine Broth, without NaAs and with NaAs (5 and 10 mg ml^–1^). The cultures were grown in 10 ml volumes in sterile Erlenmeyer flasks at 28°C for 12 h, and the turbidity at 600 nm was monitored every hour using a Tecan Infinite 200 microplate reader (Tecan, Mechelen, Belgium). Growth curves were determined for three independent cultures.

### Bioluminescence Assays

Bioluminescence assays were performed as described previously (Defoirdt and Sorgeloos, 2012). In brief, *V. campbellii* WT and mutant strains were grown overnight and diluted to an OD_600_ of 0.1. NaAs were added at 5 and 10 mg ml^–1^, respectively, the culture without NaAs was treated as control. The cultures were further incubated at 28°C with continuous shaking, and bioluminescence was measured after 1 h with a Tecan Infinite 200 microplate reader (Tecan, Mechelen, Belgium).

### Specific Quorum Sensing-Inhibitory Activity *A*_QSI_

The specific quorum sensing-inhibitory activity of NaAs at a given concentration was calculated as described previously (Yang et al., 2015):

A=Q⁢S⁢II⁢n⁢h⁢i⁢b⁢i⁢t⁢i⁢o⁢n⁢Q⁢S-r⁢e⁢g⁢u⁢l⁢a⁢t⁢e⁢dI⁢n⁢h⁢i⁢b⁢i⁢t⁢i⁢o⁢nQ⁢S-i⁢n⁢d⁢e⁢p⁢e⁢n⁢d⁢e⁢n⁢t

With

% Inhibition _QS–regulated_: percentage inhibition of QS regulated bioluminescence in wild type *V. campbellii* BAA-1116.

% Inhibition _QS–independent_: percentage inhibition of QS independent bioluminescence of *V. campbellii* JAF548 pAKlux1.

For inhibition values below 1%, a value of 1% is used in the calculations, and the *A*_QSI_ values are reported as > “calculated *A*_QSI_.” Compounds are considered as false positives if *A*_QSI_ is lower than 2 at all concentrations tested, whereas they are considered as specific QS inhibitors if they cause significant inhibition of QS-regulated bioluminescence and if *A*_QSI_ is higher than 10 at one of the concentrations tested.

### Swimming Motility Assay

The swimming motility assay was performed on soft agar (Marine agar plates containing 0.2% agar) as described previously by Rui et al. (2008). Freshly prepared and filtered NaAs (at 0, 5, and 10 mg ml^–1^, respectively) were added to the agar after autoclaving and immediately before pouring the agar into Petri plates. *V. campbellii* was grown overnight in Marine Broth, and 2 μl aliquots (OD_600_ = 1.0) were inoculated into the center of the soft agar plates. Plates were incubated for 24 h at 28°C, after which the diameters of the motility halos were measured. All assays were done with freshly prepared media in five replicates.

### Lipase and Phospholipase Assays

The lipase and phospholipase activities were measured by a modification of the method described by Austin et al. (2005). Agar plates for lipase and phospholipase assays were prepared by supplementing Marine Agar with 1% Tween 80 (Sigma-Aldrich, Belgium) and 1% egg yolk emulsion (Sigma-Aldrich, Belgium), each sterilized separately at 121°C for 5 min before mixing. The development of opalescent zones around the colonies was observed and the diameter of the zones was measured after 2–4 days of incubation at 28°C.

### Caseinase and Hemolysin Assays

The caseinase and hemolysin assays were performed as described previously with some modifications (Zhang and Austin, 2000; Austin et al., 2005). The caseinase assay plates were prepared by mixing double strength Marine Agar with a 4% skim milk powder suspension (Oxoid, Hampshire, United Kingdom), each sterilized separately at 121°C for 5 min. Clearing zones surrounding the bacterial colonies were measured after 2 days of incubation. Hemolytic assay plates were prepared by supplementing Marine Agar with 5% defibrinated sheep blood (Oxoid, Hampshire, United Kingdom) and clearing zones were measured after 2 days of incubation.

### Determination of Biofilm Levels

Biofilm formation was quantified by crystal violet staining, as described previously (Stepanovic et al., 2007). In brief, an overnight culture of *V. campbellii* was diluted to an OD_600_ of 0.1 in Marine Broth with or without NaAs, and 200 μl aliquots of these suspensions were pipetted into the wells of a polystyrene 96 well plate. Then the bacteria were allowed to adhere and grow without agitation for 24 h at 28°C. After that, the cultures were removed, and the wells were washed three times with 300 μl sterile physiological saline to remove all non-adherent bacteria. The remaining attached bacteria were fixed with 150 μl of 99% methanol per well for 20 min, after which the methanol was removed, and plates were air-dried. Then, biofilms were stained for 15 min with 150 μl per well of a 0.1% crystal violet solution (Pro-Lab Diagnostics, Richmond Hill, ON, Canada). Excess stain was rinsed off by placing the plate under running tap water, and washing was continued until the washings were free of the stain. After the plates were air-dried, the dye bound to the adherent cells was resolubilized with 150 μl of 99.8% ethanol per well, and absorbance was measured at 570 nm. Sterile medium served as a negative control, and the reported values are blank-corrected (OD_570_ of the blank was 0.1742 ± 0.006).

### Axenic Brine Shrimp Hatching

Axenic *Artemia* were obtained following decapsulation and hatching (Han et al., 2019). Briefly, 0.2 mg of *Artemia* cysts originating from the Great Salt Lake, Utah, United States (EG Type, batch 21452, INVE Aquaculture, Dendermonde, Belgium) were hydrated in 18 ml of distilled water for 1 h. Sterile cysts and nauplii were obtained via decapsulation using 660 μl NaOH (32%) and 10 ml NaOCl (50%). During the reaction, 0.22 μm filtered aeration was provided. All manipulations were carried out under a laminar flow hood and all tools were autoclaved at 121°C for 20 min. The decapsulation was stopped after about 2 min by adding 14 ml Na_2_S_2_O_3_ at 10 g l^–1^. The aeration was then terminated and the decapsulated cysts were washed with filtered (0.2 μm) and autoclaved artificial seawater (FASW) containing 35 g l^–1^ of instant ocean synthetic sea salt (Aquarium Systems, Sarrebourg, France). The cysts were suspended in 50 ml falcon tubes containing 30 ml FASW and incubated for 28 h on a rotor at 4 rpm at 28°C with constant illumination (2000 lux). The emerged nauplii at the instar II stage (at which time they start ingesting bacteria) were collected.

### Brine Shrimp Challenge Tests

The impacts of NaAs on the virulence of *V. campbellii* were determined in a standardized challenge test with gnotobiotic brine shrimp larvae. Unless mentioned otherwise, *V. campbellii* was incubated with or without NaAs at 0, 5, and 10 mg ml^–1^ concentrations for 6 h. Afterward, the cultures were washed with phosphate-buffered saline (pH 7.4) before inoculation into the brine shrimp rearing water at 10^7^ CFU ml^–1^. A suspension of autoclaved LVS3 bacteria in filtered and autoclaved seawater was added as feed at the start of the challenge test at 10^7^ cells ml^–1^. Brine shrimp cultures, to which only autoclaved LVS3 bacteria were added as feed, were used as controls. The survival of the larvae was counted 48 h after the addition of the pathogens. Each treatment was carried out in five replicates and each experiment was repeated once to verify the reproducibility. In each test, the sterility of the control treatments was checked at the end of the challenge by inoculating 1 ml of rearing water to 9 ml of Marine Broth and incubating the mixture for 2 days at 28°C.

### RNA Isolation, cDNA Synthesis, and Reverse Transcription Quantitative PCR

*Vibrio campbellii* strains were grown in triplicate in Marine Broth with or without 10 g ml^–1^ NaAs at 28°C for 24 h. Total RNA was extracted in 6 h (exponential phase) and 12 h (early stationary phase) from both groups, using RNeasy Plus Mini Kit (Qiagen, Germany) following the manufacturer’s manual. The genomic DNA was eliminated with a gDNA eliminator spin in the kit when isolating the RNA. The quality and quantity of RNA were confirmed by NanoDrop 2000 (Thermo Scientific, United States). Then an aliquot of total RNA was used to synthesize the first-strand cDNA using RevertAid H Minus First Strand cDNA Synthesis Kit (Thermo Scientific, United States) with an oligo(dT) primer. The reverse transcription quantitative PCR (RT-qPCR) assay was performed on Step One Plus Real-Time System (Applied Biosystems, United States) using Maxima SYBR Green/ROX qPCR Master Mix (2X) (Thermo Scientific, United States) in a total volume of 20 μl, containing 10 μl of 2X SYBR green master mix, 2 μl of forward and reverse primers (300 nM), and 8 μl of template cDNA. The thermal cycling consisted of an initial denaturation at 95°C for 10 min followed by 40 cycles of denaturation at 95 for 15 s and primer annealing and elongation at 60°C for 1 min. A dissociation curve analysis was performed to check for the amplification of untargeted fragments. Data acquisition was performed with the qbase plus software version 3.0 (Biogazelle, Belgium). Gene-specific primers for RT-qPCR analysis (designed by cross-exon strategy and using the software Primer Premier version 5.0) are shown in Table 1. The RNA polymerase A submit (*ropA*) was used as a reference gene (Defoirdt et al., 2007).

**TABLE 1:** Specific primers used for reverse transcriptase real-time PCR.

**Gene**	**Primer sequences (5′–3′)**	**References**
*ropA*	F: CGTAGCTGAAGGCAAAGATGA	Defoirdt et al., 2007
	R: AAGCTGGAACATAACCACGA	
*lafA*	F: TAACTTCGCATCGCTTGTAAC	VIBHAR_04961^a^
	R: TCGTCTGCTGCTGAGTTGATA	
*lafK*	F: GAGCCAAATGAACACCTCG	VIBHAR_04971^a^
	R: AACAATCGCAATCACCACA	
*LuxR*	F: ACATCAACTCAAATGGCAAGG	Rutherford et al., 2011
	R: GCAAACACTTCAAGAGCGATTT	
*pl-1*	F: ACGCCACCATCGACAAAACG	VIBHAR_02892^a^
	R: TAGGCGACCGTGGAGCATTT	
*pl-2*	F: GTCAACCGGCCAGTTATCGC	VIBHAR_06415^a^
	R: TGATCTCAACGCCTGCTGGT	
*pl-3*	F: TCGTAAGCCAACGGGTGTGA	VIBHAR_06662^a^
	R: GACGACGGCACTGCCTATTG	

**^*a*^Locus tag in the V. campbellii genome sequence (GenBank)*.*

### Statistical Analysis

Survival data were arcsine transformed to satisfy normality and homoscedasticity requirements as necessary. Data on survival, virulence factor, and bacterial growth were then subjected to one-way analysis of variances followed by Duncan’s multiple range tests using the statistical software Statistical Package for the Social Sciences version 22 to determine significant differences among treatments.

## Results

### NaAs Does Not Affect Bacterial Growth but Inhibits the QS System of *V. campbellii*

**FIGURE 2:**
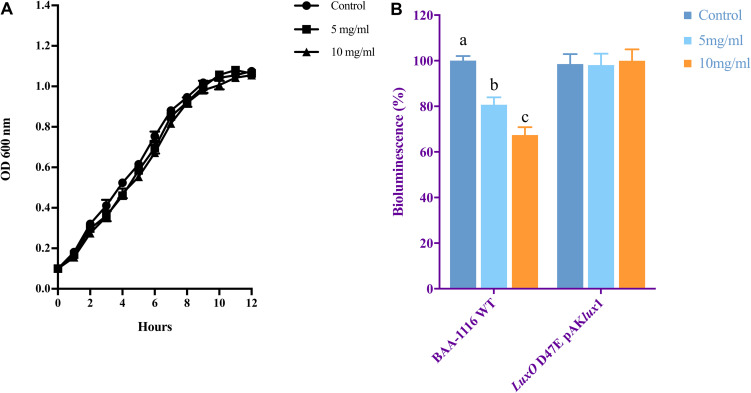
**(A)** Growth of *V. campbellii* treated with 5 and 10 mg ml^–1^ NaAs compared with untreated culture. The growth curves were determined for three independent cultures. **(B)** Impact of 5 and 10 mg m^–1^ NaAs on bioluminescence of *V. campbellii* wild type BAA-1116 and mutant *LuxO* D47E containing plasmid pAKlux1, in which bioluminescence is independent of quorum sensing. For each strain, bioluminescence in the control treatment was set at 100% and other treatments were normalized accordingly. The error bars represent the standard deviation of three replicates. Different letters indicate significant differences (One-way ANOVA with Duncan’s *post hoc* test, *P* < 0.01).

The growth of *V. campbellii* treated with 5 and 10 mg ml^–1^ NaAs (neutral pH form) was measured to verify whether indicated concentrations of this compound are bactericidal toward the strain. As shown in Figure 2A, up to 10 mg ml^–1^ NaAs did not significantly change the growth rate of *V. campbellii* as that of untreated cultures, and the early stationary stage was reached at approximately 12 h in both treated and untreated cultures. Meanwhile, NaAs was could significantly (*P* < 0.01) inhibit the production of bioluminescence in wild type *V. campbellii* at both concentrations tested (Figure 2B). To check whether the effect of NaAs on bioluminescence was due to interference with the QS, the impact of NaAs on the bioluminescence mutant strain JAF548 pAKlux1 (the bioluminescence of this strain is independent of the three-channel QS system) was measured (Defoirdt and Sorgeloos, 2012). NaAs has no effect on the bioluminescence of JAF548 pAKlux1 at the concentrations tested (Figure 2B). Consistent with this, the mRNA levels of QS master regulator *LuxR* were significantly decreased after NaAs treatment in *V. campbellii* (Table 2).

**TABLE 2:** Relative mRNA levels of lateral flagellar genes, the quorum sensing master regulator gene and phospholipase genes in *Vibrio campbellii* during incubation in Marine Broth in the absence and presence of 10 mg ml^–1^ NaAs.

	**Relative mRNA levels (fold)**			
	**6 h (exponential phase)**		**12 h (stationary phase)**	
**Gene**	**Non-treated**	**10 mg ml^–1^ NaAs**	**Non-treated**	**10 mg ml^–1^ NaAs**
*lafA*	1.00	0.28**	5.25	2.51**
*lafK*	1.00	0.19***	12.35	2.91***
*luxR*	1.00	0.56**	1.62	2.10
*pl-1*	1.00	0.48**	9.08	10.75
*pl-2*	1.00	0.23**	3.63	1.01***
*pl-3*	1.00	0.45**	3.15	18.09***

**Average standard deviation of three independent cultures*, *^∗∗^ in the graph indicates P < 0.01 and ^∗∗∗^ indicates P < 0.001 (One-way ANOVA with Duncan’s post hoc test).**

### NaAs Decreases the Swimming Motility and Production of Biofilm

**FIGURE 3:**
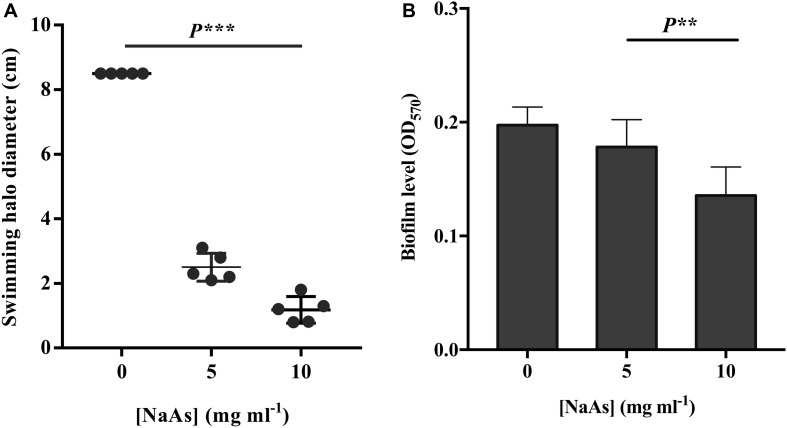
**(A)** Impact of NaAs on swimming motility of *V. campbellii* on soft agar plates. **(B)** Impact of NaAs on the biofilm levels on polystyrene 96-well plates. The error bar represents the standard deviation of five replicates for swimming motility and six independent experiments for biofilm. *P^∗∗^* in the graph indicates *P* < 0.01 and *P*^∗∗∗^ indicates *P* < 0.001 (One-way ANOVA with Duncan’s *post hoc* test).

The effect of NaAs on the swimming motility of *V. campbellii* was studied in the soft agar. NaAs was found to significantly decrease the swimming motility in a concentration-dependent manner, with a 3.4-fold decrease at 5 mg ml^–1^ and with a 7.2-fold decrease at 10 mg ml^–1^ (Figure 3). Furthermore, the effect of NaAs on the expression of selected two genes involved in flagellar motility in *V. campbellii* was investigated. The mRNA levels of the lateral flagellar regulator (*lafK*) and lateral flagellar flagellin (*lafA*) were both significantly (*P* < 0.001) decreased when comparing the NaAs-treated and untreated cells in both exponential phase and early stationary phase (Table 2). Moreover, as shown in Figure 3B, the production of the biofilm level was significantly (*P* < 0.01) affected when *V. campbellii* was treated with 10 mg ml^–1^ of NaAs.

### NaAs Decreases the Virulence of *V. campbellii* Toward Gnotobiotic Brine Shrimp Larvae

A standard challenge test with gnotobiotic brine shrimp larvae was performed to check the impact of NaAs on the virulence of *V. campbellii* toward the brine shrimp. To eliminate the effect of NaAs on the larvae, *V. campbellii* was incubated in the presence of NaAs at 5 and 10 mg ml^–1^, respectively, after which the cultures were washed to remove NaAs before adding in the rearing water. A significantly (*P* < 0.01) increased survival of brine shrimp challenged with NaAs-pretreated *V. campbellii* was observed when compared with untreated strain, indicating that the virulence of *V. campbellii* was significantly decreased after NaAs pretreatment at both the indicated doses (Table 3).

**TABLE 3:** Survival of gnotobiotic brine shrimp larvae after 2 days of challenge with *V. campbellii* BAA-1116 (average ± standard deviation of four replicates).

**Treatment**	**Survival (%)^1^**
Control	87.5 ± 2.8^c^
*V. campbellii*	45.0 ± 4.1^a^
*V. campbellii* (5 mg ml^–1^) _pretreatment_	62.5 ± 5.0^b^
*V. campbellii* (10 mg ml^–1^) _pretreatment_	70.0 ± 4.1^b^

**V. campbellii was either untreated or pretreated with NaAs prior to inoculation into the brine shrimp rearing water. “Control” refers to unchallenged larvae that were otherwise treated in the same way as the challenged larvae. ^1^Values with a different superscript letter are significantly different from each other (One-way ANOVA with Duncan’s post hoc test; P < 0.01).**

### NaAs Decreases the Production of Virulence Factors in *V. campbellii*

**FIGURE 4:**
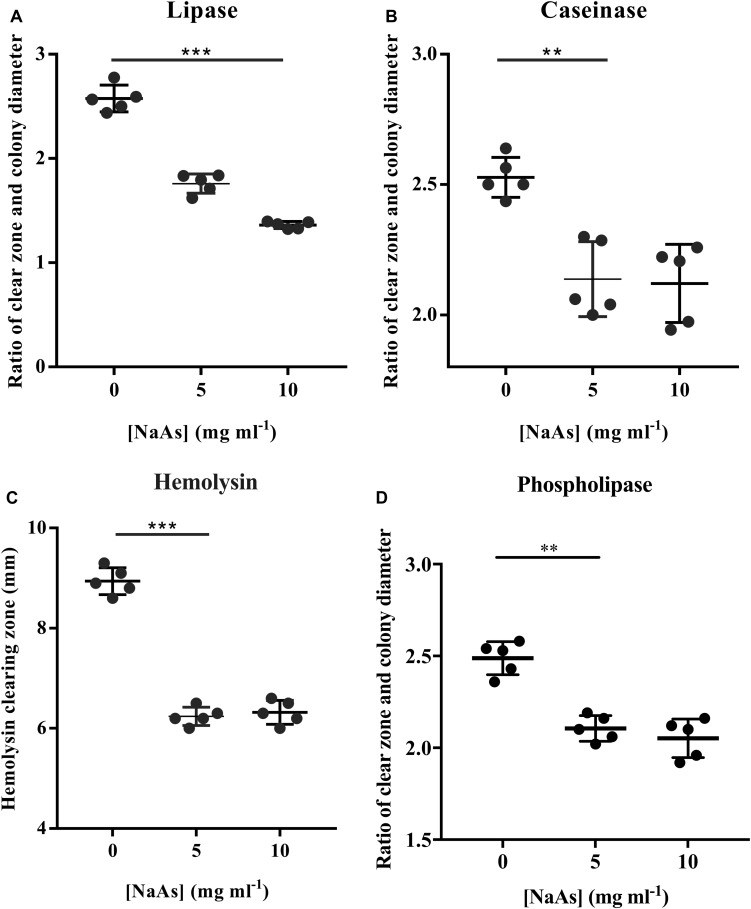
Activities of **(A)** Lipase, **(B)** Caseinase, **(C)** Hemolysin, and **(D)** Phospholipase of *V. campbellii* incubated with NaAs. The activities of the virulent determinants were expressed as the ratio of clear zone (mm) and colony diameter (mm). Results are expressed as mean ± standard error of three replicates. *P*^∗∗^ in the graph indicates *P* < 0.01 and *P*^∗∗∗^ indicates *P* < 0.001 (One-way ANOVA with Duncan’s *post hoc* test).

To determine the effect of NaAs on the production of virulence factors in *V. campbellii*, an *in vitro* study was carried out by incubating *V. campbellii* with the two doses of NaAs by plating the culture on specific agar as described above. As shown in Figure 4, a significant decrease in the activity of lipase, caseinase, hemolysin, and phospholipase were recorded due to exposure of *V. campbellii* to NaAs (5 and 10 mg ml^–1^) compared to the untreated control. The expression of three phospholipase genes was checked after *V. campbellii* treated with 10 mg ml^–1^ NaAs in both the exponential phase and early stationary phase. As shown in Table 2, the expression of three phospholipase genes was significantly (*P* < 0.01) decreased in the exponential phase compared to the untreated control. In the early stationary phase, the *pl-2* gene had the same tendency, but the expression of the *pl-3* gene was significantly (*P* < 0.01) increased.

## Discussion

In this study, an *in vivo* model system using gnotobiotically grown brine shrimp was developed to determine the impact of NaAs on the QS-regulated virulence factors in an important aquaculture pathogen *V. campbellii*. Working in the absence of other bacteria is vital for this type of experiment because it might cause bias. As far as we know, it is the first time that this type of experiment was carried out in a well-defined gnotobiotic environment. The results from the present study demonstrated that NaAs could decrease the virulence of *V. campbellii* toward the brine shrimp host, as manifested by higher survival of brine shrimp larvae exposed to the pathogen pre-treated with NaAs at either 5 or 10 mg ml^–1^ concentration (Table 3). Our results are in agreement with the findings of our previous study showing that the 20 mg ml^–1^ of halogenated furanones, (5Z)-4-bromo-5-(bromomethylene)-3-butyl-2(5H)-furanone, could increase the survival of brine shrimp larvae upon challenge with different pathogens including *V. campbellii* and *Vibrio parahaemolyticus* (Defoirdt et al., 2006). As mentioned above, many halogenated furanones are highly toxic to higher organisms (Natrah et al., 2012), and this restricts their application to control vibriosis (the diseases caused by *V. campbellii* and related vibrio pathogens) in aquaculture animals. In contrast, NaAs is relatively less toxic and well-tolerated in many farmed animals. Additionally, it is cheap, easily available, and has long been used as additives in aquaculture feeds and had proven to cause antioxidant and immune-stimulating effects in different aquaculture animals (Thomas and Holt, 1978; Waagbo et al., 1993).

However, our previous study also demonstrated that high concentration continuous exposure of NaAs (above 1 mg ml^–1^) is also toxic to brine shrimp larvae after 48 h (Han et al., 2019). Searching the optimal concentration of NaAs that decrease the virulence of the pathogen but has no adverse effect on the host would be interesting and could be applied in practice. For this purpose, the effect of 1 mg ml^–1^ NaAs (critical concentration of NaAs for toxicity in the brine shrimp culture system) on the bioluminescence of *V. campbellii* BAA-1116 wild type strain and mutant strain JAF548 pAKlux1 was measured (Supplementary Figure S1). The study showed that the non-toxic dose of NaAs could also lead to the quorum quenching effects, although the inhibition effect showed the dose-dependent manner.

Contrary to the classical antibiotics, disrupting the QS pathways of bacteria inhibits virulence rather than bacterial growth, minimizing the possibility of imposing selective pressure on the pathogen for generating resistance within the bacteria. In our study, treatment of *V. campbellii* with 5 or 10 mg ml^–1^ of NaAs showed no significant change in the growth rate when compared with untreated control. Furthermore, the density of *V. campbellii* in the brine shrimp rearing water after 1 and 2 days of the challenge was measured (Supplementary Figure S2) to verify whether the higher survival of brine shrimp larvae that were challenged with NaAs-pretreated *V. campbellii* when compared with untreated *V. campbellii* was due to the bactericidal effect of the compound. The results showed that NaAs did not exhibit bactericidal ability within the doses tested, and brine shrimp larvae in all the experimental groups were exposed to the same density of *V. campbellii*. The results demonstrated that the observed increase in the survival of *Artemia* was not due to the loss of bacterial cells. However, high concentrations of vitamin C was demonstrated to induce lethal oxidative stress via Fenton’s reaction which could sterilize the cultures of *Mycobacterium tuberculosis* (Ascorbic Acid, 4 nM) (Vilchèze et al., 2013) and *Bacillus subtilis* (Sodium ascorbate, 30 nM) (Pandit et al., 2017).

In *V. campbellii*, the density-dependent expression of the luciferase structural operon *luxCDABE* is tightly controlled by the QS system (Miller and Bassler, 2001). In this study, using the mutant strain JAF548 pAKlux1 as the control, the *A*_QSI_ value was proposed to evaluate the specific QS-disrupting activity. The *A*_QSI_ value obtained for NaAs were 20 and 33 at 5, and 10 mg ml^–1^, respectively (Figure 2B) which indicated that NaAs at these doses could cause significant inhibition of the QS-regulated bioluminescence, which is in agreement with the most specific quorum sensing inhibitor revealed previously (e.g., thiophenone TF203, *A*_QSI_ = 17 at 0.25 μM) (Yang et al., 2015). Consistent with this, the mRNA levels of quorum sensing master regulator *LuxR* were significantly decreased. Our results revealed that the downregulation of the *LuxR* and the QS-regulated phenotype (bioluminescence) has a positive correlation with the decreased virulence of *V. campbellii*, suggesting the QS-inhibitory activity largely determines the protective effect of NaAs toward the brine shrimp.

To determine the underlying virulence mechanisms in *V. campbellii*, several important virulence factors, including extracellular products (ECPs), swimming motility, and biofilm formation, were examined. The bacterial motility is an important virulence factor in many pathogens. It is essential for the pathogen during the initial phases of infection of higher organisms, including attachments, swarming, and biofilm formation (McCarter, 2001). The previous study showed that *V. campbellii* possesses dual flagellar systems, a single polar flagellum for swimming in the liquid environment while numerous lateral flagella enable the bacteria to swarm over a surface or move in other environments (McCarter, 2004). Work in our lab proved that QS positively regulates flagellar motility in *V. campbellii*, and the QS master regulator *LuxR* deletion mutant KM669 showed significantly lower motility. Moreover, the study revealed that the addition of the motility inhibitor phenamil (50 μM) could completely inhibit the swimming motility of *V. campbellii* and significantly decreased the virulence of this bacteria toward gnotobiotic brine shrimp (Yang and Defoirdt, 2015). Extending these findings, our study showed that the swimming motility of *V. campbellii* treated with NaAs was significantly impaired relative to untreated bacteria and decreased motility by NaAs has also been reported in other important pathogenic species such as *P. aeruginosa* (Abbas et al., 2012; El-Mowafy et al., 2014). The mRNA levels of the lateral flagellar regulator (*lafK*) and lateral flagellar flagellin (*lafA*) were both significantly decreased, compared with the NaAs treated and untreated cells together with the observation the decrease of the biofilm production level. Flagella are required for biofilm development at an early stage, and mutants lacking the flagellum have a decreased biofilm formation ability (Watnick and Kolter, 1999). However, the expression of the flagellar filament (*flaA*) would decrease when the bacteria cell in a mature biofilm (Moorthy and Watnick, 2004). The regulation of motility during biofilm formation in *Vibrio* species is rather complex and needs further investigation (Guttenplan and Kearns, 2013; Rossi et al., 2018).

The expression of the QS master regulator *LuxR* also significantly decreased and the previous study has also demonstrated that QS-deficient strain *P. aeruginosa* has less swimming motility and form thin and disperse biofilms (Heydorn et al., 2002). A recent study also revealed that a low concentration of vitamin C could inhibit *Bacillus subtilis* biofilm formation by deduction of extracellular polymeric substances (EPS), which is the most abundant component of the biofilm matrix and protect bacterial biofilms from hostile environmental situations (Pandit et al., 2017). Another possible explanation is that Vitamin C could inhibit the synthesis of (p)ppGpp, which is regarded as the key molecules in the stress response pathway and targeting (p)ppGpp can also inhibit biofilm production (Syal et al., 2017). Our data indicated that QS positively regulates flagellar motility and biofilm production, and NaAs can decrease the process of the colonization of the *V. campbellii* community by interference with the QS signaling pathways.

The production of extracellular virulence products (ECPs) by *V. campbellii* has been identified as one of the factors responsible for its pathogenesis, and its production is coordinately regulated by QS systems (Austin and Zhang, 2006). The increment of these virulence products may contribute to inflicting host tissue damages, allowing the pathogens to obtain nutrients and to spread through the tissues (Niu et al., 2014). In the present study, our data showed a significant reduction by NaAs of QS-related virulence factors such as lipase, caseinase, hemolysin, and phospholipase (Figure 4). Similarly, the NaAs inhibit the total protease, hemolysin, and elastase activities in *P. aeruginosa* (El-Mowafy et al., 2014). Furthermore, the expression of three phospholipase genes was checked after treatment with NaAs. The results showed that three phospholipase genes significantly decreased in the exponential phase compared to the untreated control.

## Conclusion

The present study addressed that NaAs could function as a quorum sensing inhibitor leading to decreased virulence of *V. campbellii* without affecting its growth. Moreover, NaAs was found to decrease the QS-regulated bioluminescence, suggesting the protective effect of NaAs toward the brine shrimp was due to its QS-inhibitory activity. Furthermore, NaAs can inhibit the virulence factors production, swimming motility, and biofilm formation in *V. campbellii*. These results may provide a new anti-virulence strategy of this compound for the future treatment of *Vibrio* infections in aquaculture.

## Data Availability Statement

The raw data supporting the conclusions of this article will be made available by the authors, without undue reservation, to any qualified researcher.

## Author Contributions

PB and BH designed the experiments. BH and XZ performed the experiments and analyzed the data. BH wrote this manuscript. KB, BH, and PB revised the manuscript. All authors approved the final manuscript.

## Conflict of Interest

The authors declare that the research was conducted in the absence of any commercial or financial relationships that could be construed as a potential conflict of interest.

## Footnotes

1 It should be noted here, some works of literature cited in this paper regarding the study of *Vibrio harveyi* BAA-1116 (BB120), especially for the quorum sensing study, need a change in species designation to *Vibrio campbellii* BAA-1116, according to the report from Lin et al. (2010).
