# Supplementary material for: Sodium Ascorbate as a Quorum-Sensing Inhibitor Leads to Decreased Virulence in Vibrio campbellii
Source: Front Microbiol. 2020 Jun 5;11:1054. doi: 10.3389/fmicb.2020.01054 (PMC7291813; doi:10.3389/fmicb.2020.01054)
Supplement: Supplementary file 1 [file Data_Sheet_1.docx]

Supplementary Material

The Supplementary Material include two Supplementary Figures.


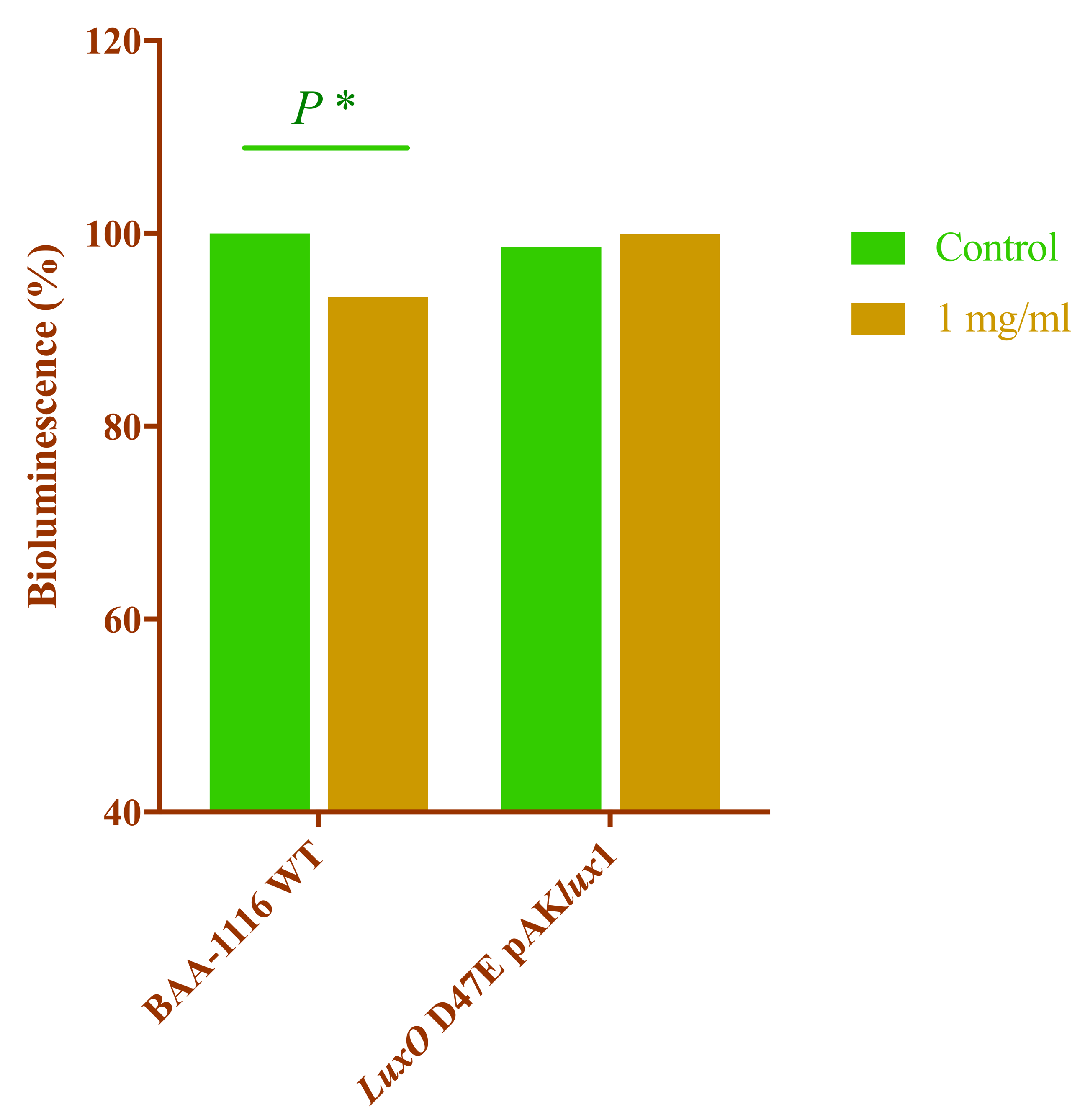


**Supplementary Figure S1.** Impact of 1 mg/ml NaAs on bioluminescence of *V. campbellii* wild type BAA-1116 and mutant *LuxO* D47E containing plasmid pAKlux1, in which bioluminescence is independent of quorum sensing. For each strain, bioluminescence in the control treatment was set at 100% and other treatments were normalized accordingly. The error bars represent the standard deviation of three replicates. Different letters indicate significant differences. (One-way ANOVA with Duncan’s *post hoc* test, *P* < 0.01).

**
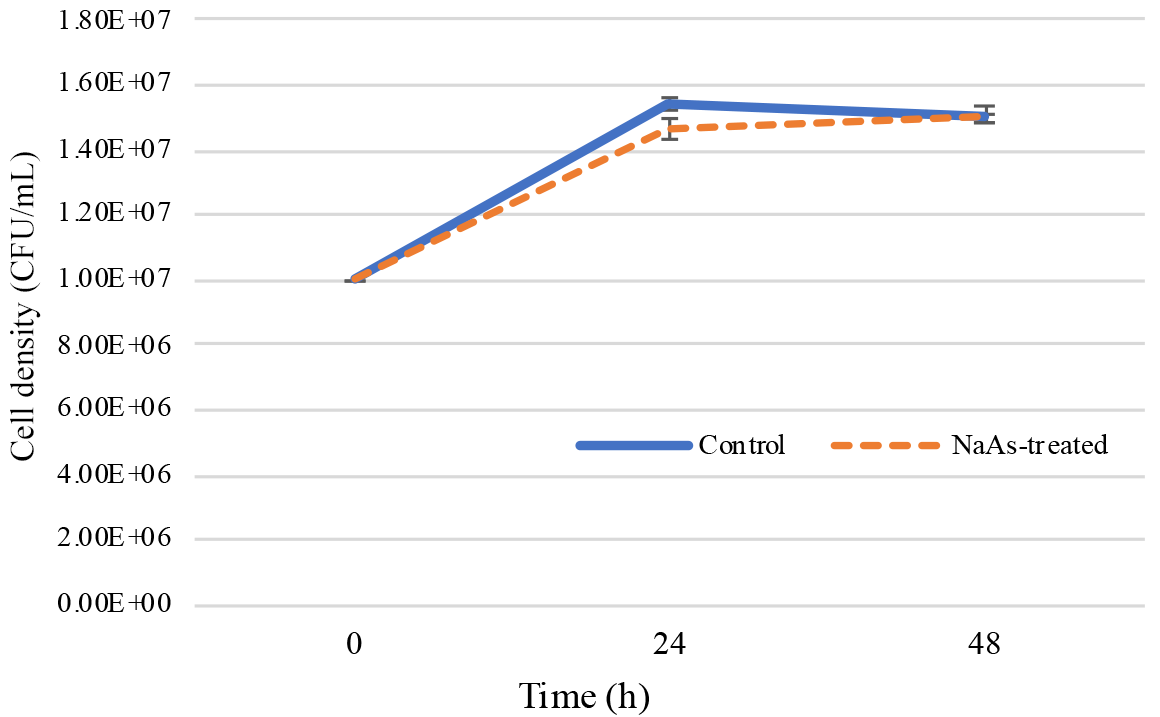
**

**Supplementary Figure S2.** Cell density of *Vibrio campbellii* in the culture water of the brine shrimp after 1 and 2 days of challenge. Error bars represent the s.d. of three replicates of cultures.
